# Silver (I) *N*-Heterocyclic Carbene Complexes: A Winning and Broad Spectrum of Antimicrobial Properties

**DOI:** 10.3390/ijms22052497

**Published:** 2021-03-02

**Authors:** Filippo Prencipe, Anna Zanfardino, Michela Di Napoli, Filomena Rossi, Stefano D’Errico, Gennaro Piccialli, Giuseppe Felice Mangiatordi, Michele Saviano, Luisa Ronga, Mario Varcamonti, Diego Tesauro

**Affiliations:** 1 Institute of Crystallography (IC) CNR, Via Amendola 122/O, 70126 Bari, Italy; Prenzfp@gmail.com (F.P.); giuseppe.mangiatordi@ic.cnr.it (G.F.M.); msaviano@unina.it (M.S.); 2 Department of Biology, University of Naples “Federico II”, via Cynthia, 80143 Naples, Italy; anna.zanfardino@unina.it (A.Z.); michela.dinapoli@unina.it (M.D.N.); 3 Department of Pharmacy and Interuniversity Research Centre on Bioactive Peptides (CIRPeB), University of Naples “Federico II”, via Mezzocannone 16, 80134 Naples, Italy; filrossi@unina.it (F.R.); stefano.derrico@unina.it (S.D.); gennaro.piccialli@unina.it (G.P.); 4 Universite de Pau et des Pays de l’Adour, E2S UPPA, CNRS, IPREM, 64000 Pau, France; luisa.ronga@univ-pau.fr

**Keywords:** NHC silver complexes, Gram-positive and negative bacteria, antimicrobial properties

## Abstract

The evolution of antibacterial resistance has arisen as the main downside in fighting bacterial infections pushing researchers to develop novel, more potent and multimodal alternative drugs.Silver and its complexes have long been used as antimicrobial agents in medicine due to the lack of silver resistance and the effectiveness at low concentration as well as to their low toxicities compared to the most commonly used antibiotics. *N*-Heterocyclic Carbenes (NHCs) have been extensively employed to coordinate transition metals mainly for catalytic chemistry. However, more recently, NHC ligands have been applied as carrier molecules for metals in anticancer applications. In the present study we selected from literature two NHC-carbene based on acridinescaffoldand detailed nonclassicalpyrazole derived mono NHC-Ag neutral and bis NHC-Ag cationic complexes. Their inhibitor effect on bacterial strains Gram-negative and positivewas evaluated. Imidazolium NHC silver complex containing the acridine chromophore showed effectiveness at extremely low MIC values. Although pyrazole NHC silver complexes are less active than the acridine NHC-silver, they represent the first example of this class of compounds with antimicrobial properties. Moreover all complexesare not toxic and they show not significant activity againstmammalian cells (Hek lines) after 4 and 24 h. Based on our experimental evidence, we are confident that this promising class of complexes could represent a valuable starting point for developing candidates for the treatment of bacterial infections, delivering great effectiveness and avoiding the development of resistance mechanisms.

## 1. Introduction

The evolution of antimicrobial resistance to most drugs pushes researchers to develop novel, more potent, and multimodal alternatives with least antibiotic effects on the human body. In this scenario, future applications can come from metal based compounds. Noteworthy, silver and its complexes have long been used as antimicrobial agents in medicine since the ancient eras [1]. Their use declined because of the penicillin discovery and the development of many other organic antibiotics. The observed resistance mechanisms led to the renaissance of silver-based antimicrobial research in the ‘60s [2] and silver based compounds in XXI century can play a relevant role in fights against infections. The success of this metal lies in the effectiveness at low concentrations and in the low toxicity. Although the action mechanism has not been fully clarified, many studies indicate that Ag(I) is the bioactive specie [3]. The lack of silver resistance could attribute to different pathways of action, such as (i) the coordination to thiol groups of cysteine residues belonging to enzymes involved in cellular respiration, or to DNA bases, (ii) the interference with electron transfer mechanism, (iii) the interaction with the cell membrane [1,2,3,4]. Evidence for the activity of the silver cations on the cell wall of the yeast *C. albicans* has been reported [5] and aqueous silver nitrate itself has antimicrobial properties. Considering that the action of silver salts is time limited complexation of silver(I) ion is desirable to increase the residence of the metal on infections. One of the crucial key factors decisive for magnitude of antimicrobial properties of silver complexes is the capability to generate silver (I) ion that interacts with biological ligands (e.g., proteins) [4]. Consequently, the nature of the atom coordinated to the metal center and its bonding properties play a decisive role, rather than the chirality of the ligands, solubility, charge, or degree of polymerization of the silver complexes.

Furthermore, the solubility and stability of silver complexes in chloride solution are key factors that limit their use for in vivo applications [6].

After the isolation of the first benzimidazolecarbene by Arduengo in 1991 [7], *N*-Heterocyclic Carbenes (NHCs) ligands have been employed to coordinate, due to their easy synthetic procedures and the stability of the carbene center brought by two adjacent nitrogen atoms [8,9]. 

Metal-NHC complexes have been primarily used in catalytic chemistry [10,11]. However, more recently NHC ligands have been applied as carrier molecules for metals in anticancer applications [12]. The reported number of NHC-metal complexes is continually growing and several reviews have been published [13,14]

In this context, silver(I)-*N*-Heterocyclic Carbene (Ag(I)-NHC) complexes can find their role. The antimicrobial and anticancer properties of this class of complexes have been reviewed in the last decade [14,15]. The main goal in designing antimicrobial Ag-NHC is to achieve slow dissociation of Ag^+^ from Ag-NHC in the affected area, retaining the effectiveness on the wound-site. Actually, the activities of the NHC–silver complexesare considerably affected by the NHC ligand structures [13]. The factors such as hydrophobic substitution and steric bulk on imidazole ring can delay the rate of silver ions release [16]. Tacke’s group demonstrated that introducing lipophilic benzyl-substituents at the *N*1 and *N*3 positions, starting from 4,5-diphenylimidazol the resulting silver complexes show a minimum inhibitory concentration (MIC), ranging from 20 to 3.13 µg/mL (35.3 to 5.52 µM) for a variety of Gram-positive, Gram-negative and mycobacteria tested [17]. Only Haque et al. reported a compared study, synthesizing a series of mono- and binuclear silver(I) complexes [18]. All binuclear complexes having general formula [NHC-Ag-NHC]_2_2PF_6_, and mono-carbene of general formula [NHC-Ag-Br] showed antimicrobial activity. Mononuclear complexes displayed three-fold lesser activity in the same biological conditions against the same bacterial strains.

**Scheme 1:**
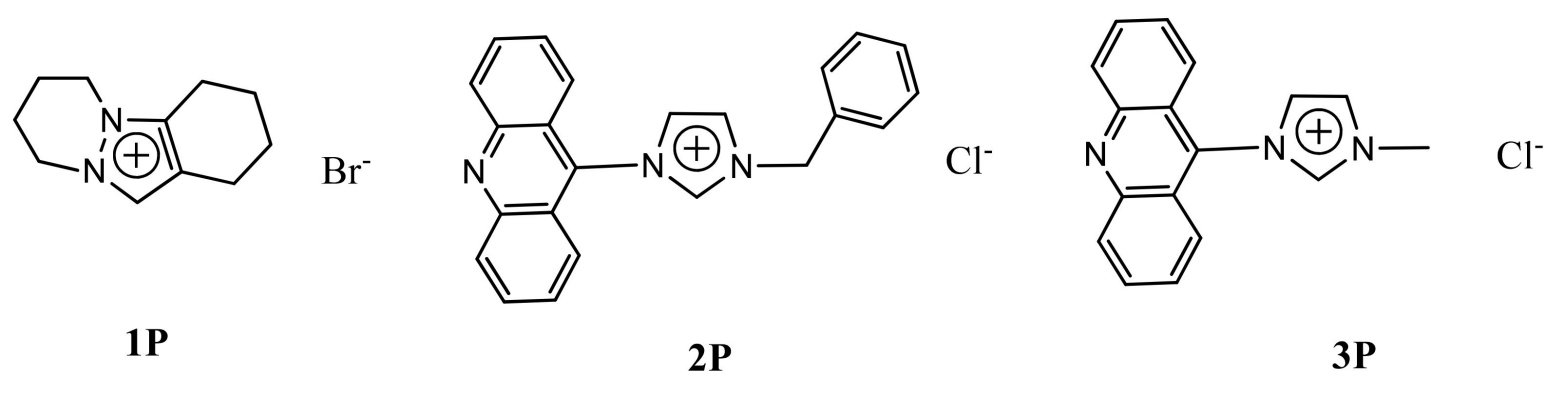
2D structures of the investigated ligands.

**Scheme 2:**
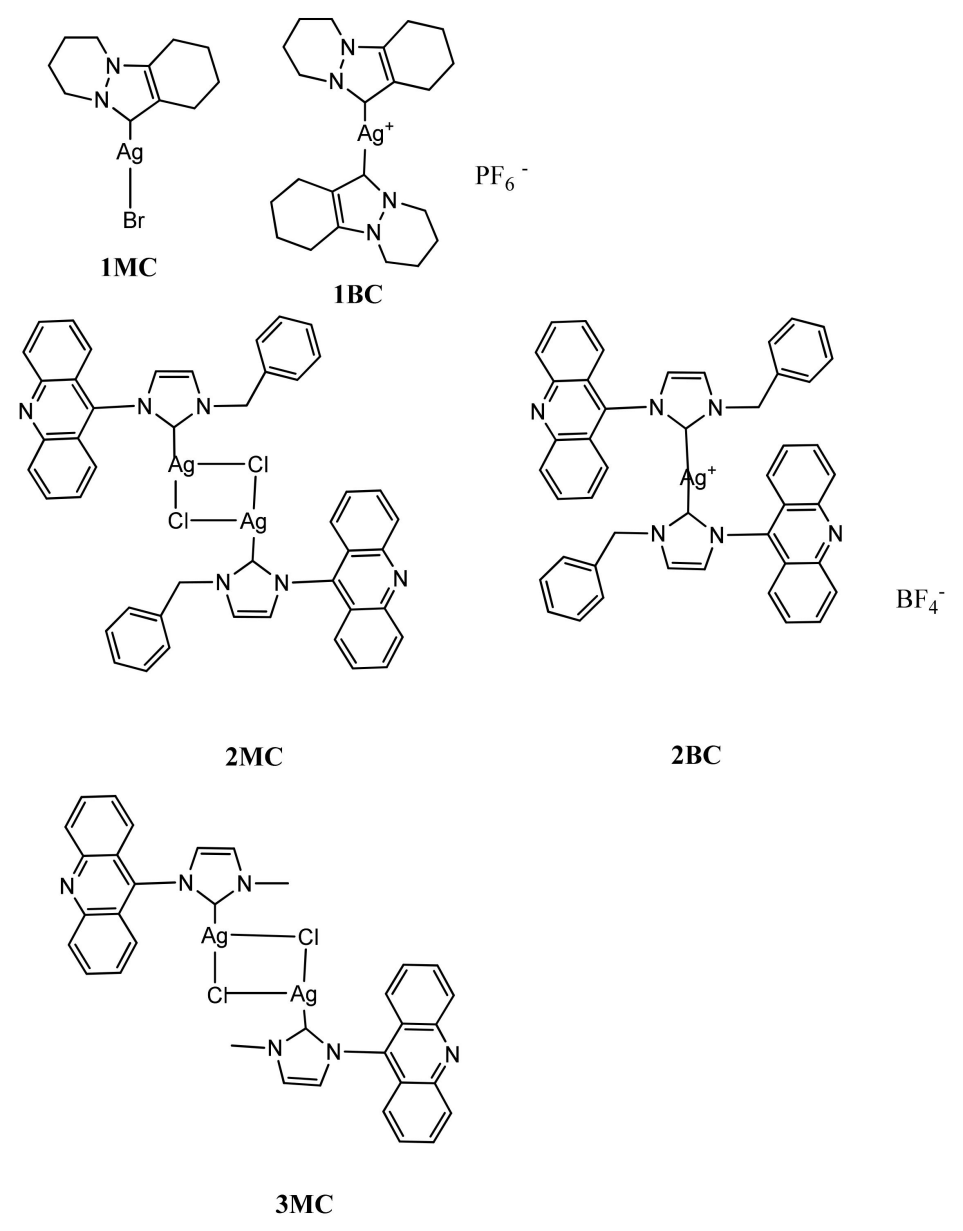
2D structures of the investigated silver complexes.

In our study, we selected from literature two NHCs based on acridine [19]. This moiety is an excellent candidate as antimicrobial ligand. Moreover, the optical properties of acridine-NHC ligands allow to monitor their presence on the bacteria cells. The aim was to study the inhibitor effect on bacterial strains Gram-negative and positive of neutral mono NHC-Ag complexes and cationic bis NHC- Ag complexes. Furthermore, we tested and detailed non-classicalpyrazole derived mono NHC-Ag neutral and bisNHC-Ag cationic complexes. For all compounds listed in Scheme 1 and Scheme 2, we focused our attention on the effect of exposition to the light and belonging fluorescence assets into bacterial cells after the treatments.

## 2. Results

### 2.1. Design and Synthesis

Free acridine bearing imidazole carbene ligands (2P, 3P) and their silver complexes (2MC, 2BC, 3MC), wereselected among different reported literature silver complexes. In particular, we focused our attention on those compounds characterized by synthetic accessibility and an octanol/water partition coefficient (logP) compatible with a good membrane permeability, being the computed values ranging from 2.21 (1MC) to 8.87 (2BC) (Table 1). These hydrophobic compounds are potentially able to cross cell membrane and to produce π-π stacking interactions with DNA bases. The synthesis and physico-chemical properties of mono NHC (2MC-3MC) and bis NHC (2BC) silver complexes were well described and known [19]. The syntheses were carried out following the reported procedure and the results were compared to yet reported data.

A reported X ray structure of 3MC demonstrated that two molecules are associated around an inversion center, giving dimeric structures with lateral metal-chlorido interactions [19].

The pyrazolecarbene ligands were designed following the same criteria. The pyrazolecarbene ligand exerts a higher π-donor/σ-acceptor ratio than imazolin-2-ylidenes [20]. The mono NHC and bis NHC silver complex (1MC-1BC) were synthesized adapting well assessed procedures for imidazolium carbene ligands by adding the respectively cationic ligand to Ag_2_O in dichloromethane and by stirring the mixture for 6 h at room temperature. Bis-carbene complexes were obtained by adding two equivalents of ligand, when the anionic group does not have coordination ability.

After crystallization, structures of all compounds were identified by ^1^H NMR, ^13^C NMR and ESI MS experiments (See Supplementary Materials).

### 2.2. Biological Assays

MICs determination was performed against *Escherichia coli* to test the antimicrobial activity of silver-containing mono- and bis-carbenes, along with their ligands (Table 2). In comparison to silver salts—already known for their strong antibacterial activity—mono-carbene silver complexes show very low MIC values.

Among the bis-carbene complexes, 2BC shows a strong antimicrobial activity while compared to the other complex of the same family. The ligands—i.e., 1P, 2P and 3P—do not show a significant antimicrobial activity, thus confirming the hypothesis of a connection between the Ag ions and the antimicrobial activity. To get deeper into the biological analysis, we choose samples showing a MIC value less or equal to 1 μM, and we widen the indicator strains panel with two Gram-negative (*E. coli* and *P. aeruginosa*) and two Gram-positive bacteria (*B. subtilis* and *S. aureus*). MIC values against all the considered strains were quite comparable (Table 2).

**Figure 1:**
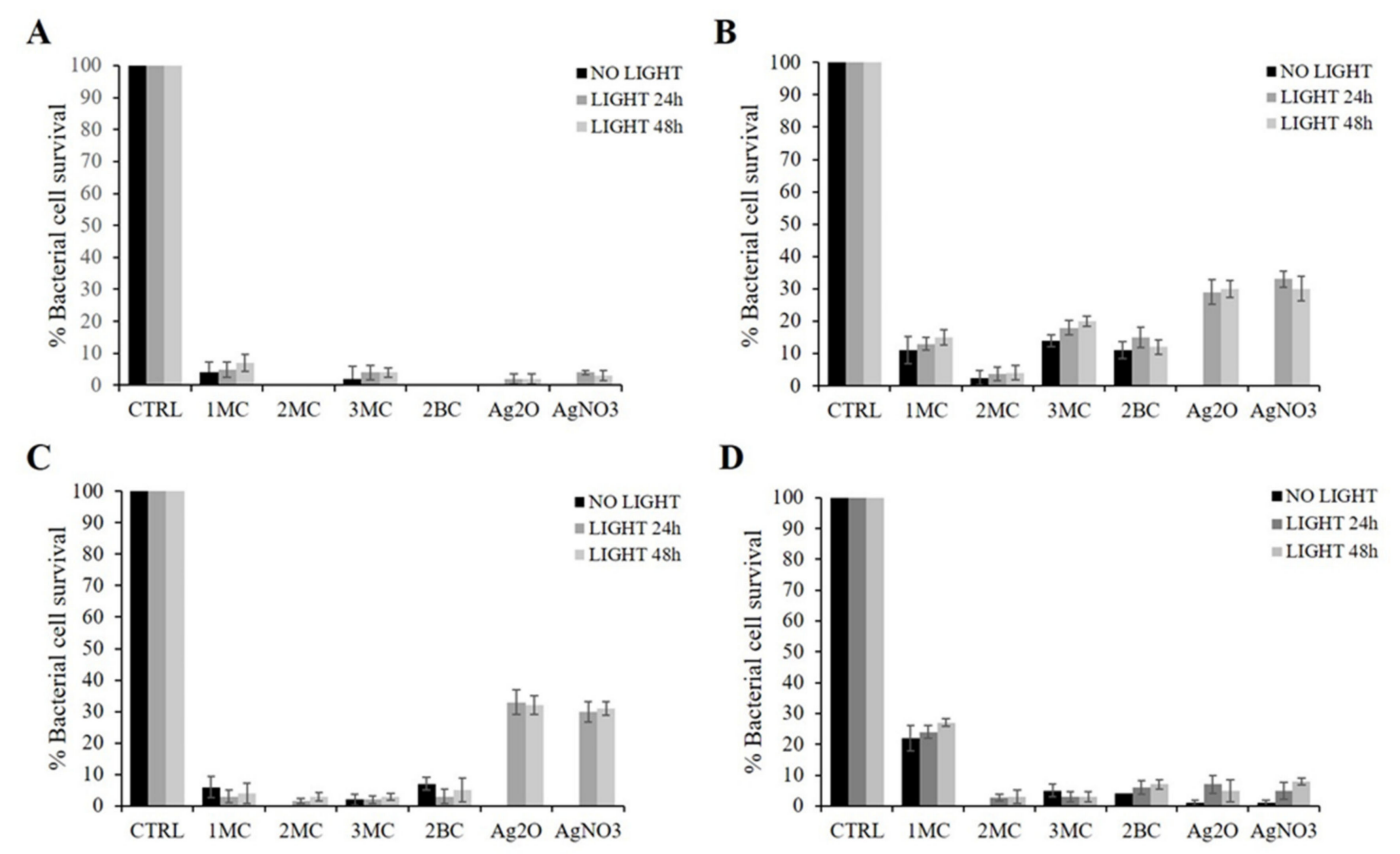
Antibacterial activity of 1MC, 2MC, 3MC, 2BC, Ag_2_O and AgNO_3_, exposed or not to light for 24 and 48 h evaluated by colony count assay, against *E. coli* DH5α (panel **A**), *S. aureus* ATCC 6538P (panel **B**), *P. aeruginosa* PAOI (panel **C**), and *B. subtilis* PY79 (panel **D**) at a fixed concentration of 1μM. Untreated cells represented negative control. Each bar is calculated as the average of three different experiments. *p* value is < 0.05.

In order to test the stability of the antibacterial molecules we exposed to visible light mono-carbenecomplexes, the 2BC bis-carbene complex and two silver salts for 4, 24 and 48 h. Also after 48 h of light exposition 2MC, 3MC and 2BC complexes, were still active, while Ag salts reduced their activity at least against *P. aeruginosa* and *S. aureus*, as shown in Figure 1.

**Figure 2:**
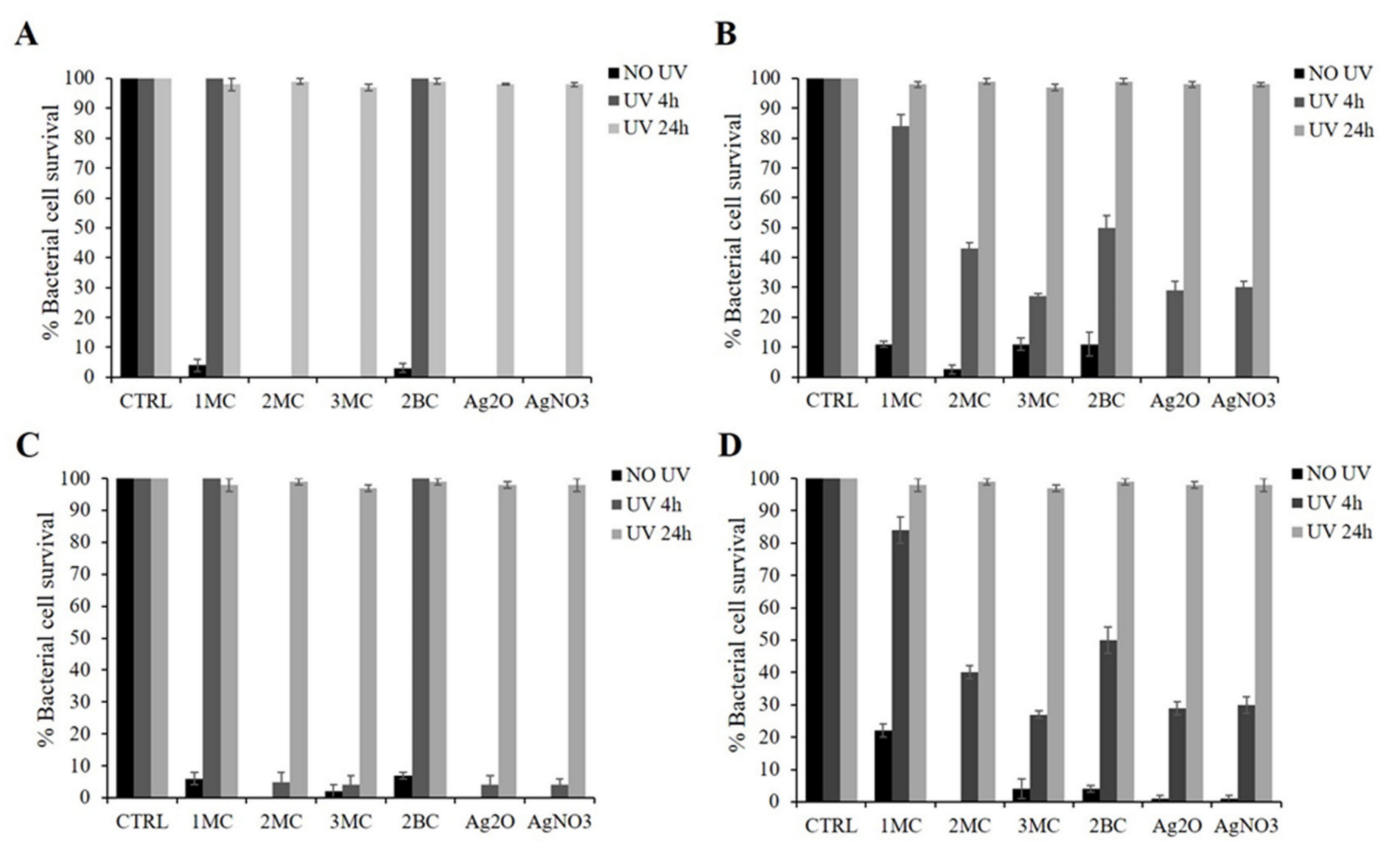
Antibacterial activity of 1MC, 2MC, 3MC, 2BC, Ag_2_O and AgNO_3_, exposed or not to UV for 4 and 24 h evaluated by colony count assay, against *E. coli* DH5α (panel **A**), *S. aureus* ATCC 6538P (panel **B**), *P. aeruginosa* PAOI (panel **C**), and *B. subtilis* PY79 (panel **D**) at a fixed concentration of 1μM. Untreated cells represented negative control. Each bar is calculated as the average of three different experiments. *p* value is < 0.05.

We also measured the antimicrobial activity of different complexes after UV light (254nm) exposition (Figure 2). Antibacterial activity of 1MC and 2BC were strongly reduced already after a treatment of 4 h. UV treatment of 2MC, 3MC and Ag salts for 24 h completely inactivated their antimicrobial capacity, while after 4 h of UV exposure they still exert a significant antimicrobial activity against the target bacterial strains.

The 2MC complex is able to emit fluorescence detectable with a TRITC filter (excitation/emission: 550/570nm). Thanks to this feature, we analyzed by fluorescence microscopy the interaction of 2MC with *E. coli*.

**Figure 3:**
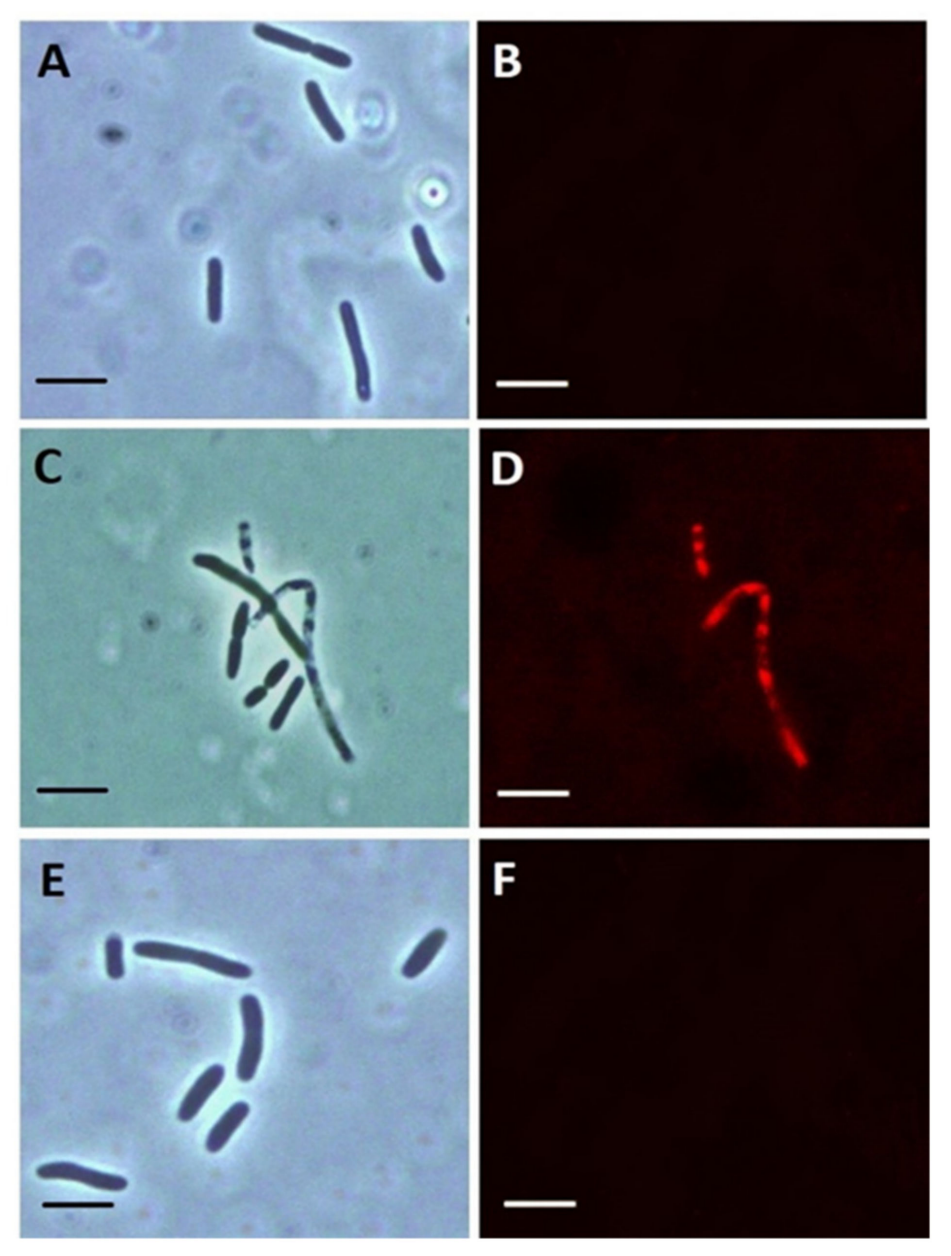
Panels show *Escherichia coli* DH5α bacterial cells observed in optical microscopy (**A**,**C**,**E**) and in fluorescence microscopy (**B**,**D**,**F**). Untreated bacterial cells (**A**,**B**); cells treated with 2MC (**C**,**D**), cells treated with 2P (**E**,**F**). Scale bars: 1 µm (**A**–**F**).

As shown in Figure 3, panels A and B, bacterial cells used as control appeared intact and dark gray in optical, phase contrast, microscopy (panel A), and do not develop any fluorescence (panel B). Cells treated with 2MC appeared altered in shape and color, as shown in optical, phase contrast, microscopy (Figure 3, panel C), and panel D, shows a clear red fluorescence indicating significant interaction between bacterial cells and 2MC. To confirm the role of NHC-silver complex as antimicrobial agent, we incubated *E. coli* cells with 2P. In Figure 3 panel E, the cells treated with 2P appeared similar to the control and in panel F, they do not present fluorescence signals. All compounds were tested against mammalian cells (Hek lines). They show little (after 24h) or no (after 4h) toxicity as shown in Figure S1 (see Supplementary Materials).

## 3. Discussion

In our study, we selected from literature NHC silver complexes focusing on their potential ability to cross bacterial cell membrane. In particular2P and 3P ligands are based on acridine [19], an outstanding candidate as antimicrobial ligand.As well demonstrated by Bierbach et al. [21] some gold(I) complexes bearing this ligand display selective antimicrobial activity against *Mycobacterium tuberculosis* acting as carrier ligand or as target agent.Furthermore, acridinebased complexes may be used on blood samples causingbacterial DNAto fluoresce, aiding in the clinical diagnosis of bacterial infections (e.g., meningitis). The high effectiveness of asymmetrical acridine NHC silver complexes may well suit to synergistic effects due to hybridization of two bioactive agents. Above all, the site of action of acridine derivatives is the bacterial nucleic acid, being facilitated the intercalation by molecular planarity and cationic ionization. This last assessed joined with high hydrophobicity increases the amount of internalization of silver complexes. Both asymmetrical acridine NHC-silver complexes show a similar effect on all bacterial strains; benzyl and methyl moieties are not decisive for the effectiveness. 2MC and 3MC mono-carbene complexes show at least the same activity resulting from thebis-carbene cationic one. These results could depend on the greater stability of mono NHC complexes that could be favor its internalization in the prokaryotic cells. Another hypothesis could be advanced: the AgCl_2_^−^ species, considered the bioavailable form in the bacterial cell preventing the formation of high insoluble AgCl salt [22], it is already formed in the dimeric complex form. Furthermore, the stability to the light exposition of acridine imidazolium NHC silver complexes is verified after 48 h.The antimicrobial activity of this class of compounds is preservedbetter than silver salts. Fluorescence images (Figure 3) allow us to demonstrate the interaction with *Escherichia coli* of the 2MC silver complex after 1 h of incubation. Notably, fluorescence emission could be only detected after incubation with 2MC complex wherethesame incubation with 2P ligand do not show fluorescence emission at all. This result testifies that the complex retains its integrity during the interaction with bacteria cells and that only damaged bacteria emit fluorescence, this observation allows us to establish a cause and effect principle between the action of the compound and the death of the bacterium.

In literature pyrazole NHC ligands were synthesized only in ancillary moiety of imidazolium NHC-silver complexes [23,24] coordinating the metal center through N atom. Only one example of pyrazole-NHC gold complex is reported [25]. To the best of our knowledge, these complexes are first silver complexes with pyrazoleNHCs.Thepyrazole NHC ligand due to his higher σ-donor/π-acceptor ratio compared to imazolin-2-ylidenes increases the density charge on metal center stabilizing both complexes. We have also observed less efficacy of pyrazole NHC silver complexes although the MIC is still very low. This minor effectiveness could be attributed to lower hydrophobicity with respect to the acridine based ligand complexes as indicated by the computed log *p*.

## 4. Experimental

### 4.1. Materials and Physicochemical Measurements

Solvent and Chemical reagents were purchased commercially and used as received without further purification; (Sigma-Aldrich, Steinheim, Germany), 1H NMR spectra were acquired with Brucker (Billerica, MA, USA))400 MHz, ESI mass spectra were recorded in positive mode with an Applied Biosystems(Foster City, CA, USA) mass spectrometer equipped with a triple quadrupole mass analyzer. The [(BenzylImAcr)H]Cl (2P), [(BenzylImAcr)H]BF_4_, [(CH_3_ImAcr)H]Cl (3P), [CH_3_ImAcr]BF_4_, NHC ligands were obtained following the described procedure [19,25]. 2MC, 3MC; 2BC complexes were synthesized adapting reported methods [19]. Selected spectra are reported in the Supplementary Materials. The 2D structures of the complexes under investigation were drawn using ChemDraw Professional (version 17) (https://pubs.acs.org/doi/full/10.1021/ja0697875 accessed on 20 January 2021) and exported to a .sdf file. This file was imported into Canvas [26], a cheminformatics package useful for calculating molecular properties starting from 2D structures and available from the Schrödinger suite. In particular, the octanol/water partition coefficient (logP) was calculated for each complex using the AlogP approach [27]. All values are listed in Table 1.

### 4.2. Synthesis of Ligand (1,2,3,4,6,7,8,9-octahydropyridazino [1,2-a]indazolin-11-ylidene), (FPyr·HBr) (1P)

1P was prepared with a modified procedure previously reported [25]. NaOH (136 mg, 3.4 mmol) was added to a solution of 4,5,6,7-tetrahydro-1-H-indazole (244 mg, 2 mmol) in acetonitrile (30 mL), and the resulting mixture was stirred for 2 h at room temperature. 1,4-Dibromobutane (427 μL, 3.56 mmol) was then added and the reaction mixture was stirred for a further 2 h at room temperature. Subsequently, the reaction mixture was heated at 90° C and refluxed for 24 h. The solvent was removed under vacuum, and the residue obtained was suspended in CH_2_Cl_2_ and filtered. The filtrate was concentrated and the oily residue obtained was purified by column chromatography on silica gel (CH_2_Cl_2_/MeOH 9:1) affording the product as a off-white powder (330 mg, 1.28 mmol, 64%).^1^HNMR (400MHz, CDCl_3_) δ 8.38 (s, 1H), 4.72 (t, *J* = 5.8 Hz, 2H), 4.42 (t, *J* = 6.0 Hz, 2H), 2.79 (t, *J* = 6.3 Hz, 2H), 2.59 (t,*J* = 6.0 Hz, 2H), 2.29 (m, 4H), 1.92 (m, 2H), 1.81 (m, 2H).ESI^+^(MS) = m/z 177 [M^+^]

### 4.3. Synthesis of FpyrAgBr (1MC)

Ag_2_O (16 mg, 0.069 mmol) was added to a stirring solution of 1P (29 mg, 0.112 mmol) in CH_2_Cl_2_ (15 mL) and the resulting mixture was stirred for 6 h at room temperature, shielded from light. The reaction mixture was filtered through a pad of Celite, and the solvent of the filtrate was concentrated under vacuum and the product was precipitated by slow infusion of diethyl ether. The product was obtained as a cream solid (17 mg, 0.046 mmol, 42%). ^1^H NMR (400 MHz, DMSO d_6_) δ 4.28 (bt, 2H, NCH_2_), 4.06 (bt, 2H, NCH_2_), 2.60 (t, 2H,*J* = 6.1 Hz, CH_2_), 2.46 (t, 2H,*J* = 5.9 Hz, CH_2_), 2.02 (m, 4H, 2x CH_2_), 1.78 (m, 2H, CH_2_), 1.69 (m, 2H, CH_2_). ^13^C NMR (100 MHz, DMSO d_6_) δ 176.31 (1C, AgC), 142.84 (1C, sp^2^C), 125.29 (1C, sp^2^C), 52.06 (1C, CH_2_N), 45.85 (1C, CH_2_N), 23.29 (1C, CH_2_), 23.24 (1C, CH_2_), 22.20 (1C, CH_2_), 21.81 (1C, CH_2_), 20.53 (1C, CH_2_), 20.39 (1C, CH_2_). ESI^+^ (MS) = m/z 177.1 [M–Ag–Br]. Anal.Calc (%) for C_11_H_16_N_2_BrAg: C,36.29%; H,4.43%; N,7.70% Found:C, 36.43%; H, 4.38%; N, 7.62%. 

### 4.4. Synthesis of [(Fpyr)_2_Ag]BF_4_ (1BC)

The complex was synthesizes adapting a well assessed procedure previously reported [25]. Hexafluorophosphate salt of 1P (35 mg, 0.11 mmol) tetrabutylammonium bromide (35 mg, 0.11 mmol) and Ag_2_O (26 mg, 0.11 mmol) were stirred in CH_2_Cl_2_ (20 mL) for 6 h at room temperature shielded from light. The reaction mixture was filtered through a pad of Celite, the solvent of the filtrate was concentrated under vacuum and the product was precipitated by slow infusion of diethyl ether. The product was obtained as an off-white solid (30 mg, 0.049 mmol, 45%). ^1^H NMR (400 MHz, DMSO d_6_) δ 4.28 (bt, 4H, 2CH_2_, NCH_2_), 4.06 (bt, 4H, 2CH_2_, NCH_2_), 2.60 (bt, 4H, 2CH_2_), 2.47 (bt, 4H, 2CH_2_), 2.02 (m, 8H), 1.78 (m, 4H), 1.70 (m, 4H). ^13^C NMR (100 MHz, DMSO d6) δ 176.36 (2C, 2 AgC), 142.84 (2C, 2 sp^2^C),125.31(2C, 2 sp^2^C), 52.06 (2C, 2 CH_2_N), 45.84 (2C, 2 CH_2_N), 23.29 (2C, 2 CH_2_), 23.23 (2C, CH_2_), 22.20 (2C, CH_2_), 21.81 (2C, CH_2_), 20.52 (2C, 2x CH_2_), 20.38 (2C, CH_2_). ESI^+^ (MS) = m/z 459Anal.Calc% for C_22_H_32_N_4_PF_6_Ag: C, 43.65%; H, 5.33%; N, 9.26% Found: C, 43.84%; H, 4.31%; N, 7.51%.

### 4.5. Minimum Inhibitoryconcentration (MIC)

To determine the minimal inhibitory concentration (MIC), assays were performed as previously described elsewhere [28,29] with different ligands (1P, 2P, 3P) complexes: (1MC, 2MC, 3MC, 1BC, 2BC) and salts Ag_2_O and AgNO_3_. Briefly, bacteria were grown to mid-logarithmic phase at 37 °C and then diluted to 1 × 10^6^ CFU/mL in Difco 0.5 × Nutrient Broth (Becton-Dickenson, Franklin Lakes, NJ) containing increasing amounts of compounds (0.5–50 μM). Starting from a compounds stocks solution, two-fold serial dilutions were sequentially carried out, accordingly to broth microdilution method [30]. Following over-night incubation, MIC100 values were determined as the lowest concentration associated with absence of bacterial growth.

### 4.6. Antimicrobial Assays

Antimicrobial activities of 1MC, 2MC, 3MC, 2BC, Ag_2_O and AgNO_3_ moleculeswere valuated against two Gram-negative bacteria: *Escherichia coli* DH5α and *Pseudomonas aeruginosa* PAOI and two Gram-positive bacteria: *Staphylococcus aureus* 6538P and *Bacillus subtilis* PY79. A single colony of each strain was suspended in 5 mL of Luria-Bertani (LB) broth and incubated overnight at 37 °C. When the culture reached an OD600 of 1 unit, it was diluted 1:100 in 20 mMPBS buffer at pH 7.0. Samples were prepared by adding 1/25 of the volume of bacterial cells and the compounds were used at concentrations of 1 μM, 500 μL final volume was reached with 20 mMPBS buffer at pH 7.0 [31]. Negative control was represented by cells with any treatment. Samples were incubated at 37 °C for 4 h in the dark, two dilutions (1:100 and 1:1000) of all the samples were placed on solid medium LB agar and incubated overnight at 37 °C. The same assay was performed by exposing the complexes to light for 24 and 48 h or to UV (254nm) for 4 and 24 h before incubation with the cells of each strain. The following day, the surviving cells were estimated by colony counting on each plate and compared with the controls [32]. Standard deviations were less than 5% for each experiment (which was performed at least in triplicate).

### 4.7. Fluorescence Microscopy Image Acquisition

To evaluate the fluorescence, 50 μL of *Escherichia coli* DH5α (bacteria were grown to mid-logarithmic phase) were incubated for 1 h at 37 °C in the presence or/and absence of 2MC and 2P at fixed concentration of 1μM. After the incubation, 10μL of both samples were observed using an Olympus BX51 fluorescence microscope (Olympus, Tokyo, Japan) using a tetrametil-rodamina-isotiocianato (TRITC) filter (excitation/emission: 550/570 nm). Standard acquisition times were 1000 ms for staining. Images were captured using an Olympus DP70 digital camera.

### 4.8. Eukaryotic Cell Cultures

HEK 293 (human embryonic kidney) is a widely used in scientific research specific cell line originally derived from human embryonic kidney cells. Cells were maintained in Dulbecco Modified Eagle Medium (DMEM), supplemented with 10% fetal bovine serum and 1% penicillin streptomycin. Cells were cultured at 37 °C in humidified atmosphere of 5% CO_2_. The compounds 1P, 2P, 3P, 1MC, 2MC, 3MC, 1BC, 2BC, Ag_2_O e AgNO_3_ were used for the cytotoxicity assay [33].

### 4.9. Cytotoxicity Assay on Mammalian Cells

Cytotoxicity on HEK 293 cells was assessed by performing the 3-(4,5-dimethylthiazol-2-yl)-2,5 diphenyltetrazolium bromide (MTT) reduction inhibition assay, the colorimetric assay for assessing cell metabolic activity. Cells were grown as previously described and plated on 96-well plates at a density of 5 × 10^3^ cells per well, in 200 μL of medium containing compounds at concentration of 1 μM for 4 and 24 h. After 4 and 24 h of treatment, the medium was aspirated and 10 μL of a stock MTT solution was added to the cells to a final concentration of 0.5 mg/mL. After 4 h incubation the MTT solution was removed and the formazan salts were dissolved in 100 μL of 0.1 N HCl in anhydrous isopropanol. Cell survival was expressed as the absorbance of blue formazan measured at 570 nm with an automatic plate reader (Multi scan spectrum, Thermo Scientific, Waltham, MA, USA). Cytotoxicity test was performed at least 3 times. Standard deviations were always <5% for each experiment [34].

## 5. Conclusions

Imidazolium NHC silver complexes containing the acridine chromophoreshave been synthesized and tested on two Gram-negative bacteria: *Escherichia coli DH5α* and *Pseudomonas aeruginosa* PAOI and two Gram-positive bacteria: *Staphylococcus aureus* 6538P and *Bacillus subtilis* PY79. 2MC, 2BC, 3MC complexes have shown effectiveness at extremely low MIC values. This class of complexes is active at concentration 100 times lower than NHC silver complex tested in literature [15]. Moreover, they are resistant to light stress and, for a short time, giving better performance than silver salts. We tested for the first time pyrazole NHC silver complexes. The different electronic properties of this ligand do not induce the loss of antimicrobial activity. Finally these compounds showed not significant toxicity against mammalian cells. Further experiments are needed to identify the action mechanism of these compoundsprecisely. However, based on our experimental evidence, we are confident that these promising trends will be usedfor this class of complexes by new and specific development tags as valid candidates in antimicrobials applications.

## Figures and Tables

**Table 1:** Computed log *p* values of the selected complexes.

Complexes	Log *p*
1MC	2.21
2MC	8.66
3MC	5.60
1BC	4.41
2BC	8.87

**Table 2:** Mono-carbene silver complexes (1MC, 2MC and 3MC), bis-carbene silver complexes (1BC and 2BC), ligands (1P, 2P and 3P), and silver salts MIC values against different strains.

*E. coli* DH5α		*P. aeruginosa* PAOI		*B. subtilis* PY79		*S. aureus* 6538P	
Compound	MIC[μM]	Compound	MIC[μM]	Compound	MIC[μM]	Compound	MIC[μM]
1P	>50	1P	>50	1P	>50	1P	>50
2P	>50	2P	>50	2P	>50	2P	>50
3P	>50	3P	>50	3P	>50	3P	>50
1MC	5	1MC	10	1MC	10	1MC	10
2MC	≤1	2MC	≤1	2MC	≤1	2MC	≤1
3MC	≤1	3MC	≤1	3MC	≤1	3MC	≤1
1BC	25	1BC	40	1BC	40	1BC	50
2BC	≤1	2BC	≤1	2BC	≤1	2BC	≤1
Ag_2_O	≤1	Ag_2_O	≤1	Ag_2_O	≤1	Ag_2_O	≤1
AgNO_3_	≤1	AgNO_3_	≤1	AgNO_3_	≤1	AgNO_3_	≤1

## Supplementary Materials

Supplementary materials can be found at https://www.mdpi.com/1422-0067/22/5/2497/s1.
