# Supplementary material for: Silver (I) N-Heterocyclic Carbene Complexes: A Winning and Broad Spectrum of Antimicrobial Properties
Source: Int J Mol Sci. 2021 Mar 2;22(5):2497. doi: 10.3390/ijms22052497 (PMC7958610; doi:10.3390/ijms22052497)
Supplement: Supplementary file 1 [file ijms-22-02497-s001.pdf]

Silver (I) *N*-Heterocyclic carbene complexes: A winning and broad spectrum of antimicrobial properties

Filippo Prencipe, Anna Zanfardino,Michela Di Napoli, Filomena Rossi, StefanoD'Errico, Gennaro Piccialli, Giuseppe Felice Mangiatordi, Michele Saviano, Luisa Ronga, Mario Varcamonti, Diego Tesauro

Analitical data of the reported compounds [19]:

$[AgCl(PhCH_2imAcr)]$ (**2MC**). ^1H NMR (DMSO-d_6,400 MHz, 298 K): δ 8.22 (br, 2H, acr), 7.86 (br, 2H, acr),7.45 (br, 2H, acr), 7.31-7.21 (br, 2H, acr and 3H, Ph), 6.93 (br, 2H, Ph), 4.93 (br, 2H, CH_2). ^{13}C NMR (DMSO-d_6,100.6 MHz, 298 K): δ 179.1 (im),148.4 (acr), 139.5 (acr), 136.2 (Ph), 130.8(acr), 129.3 (Ph), 128.7 (acr), 128.0 (Ph), 127.9 (Ph), 127.3 (acr), 125.6 (im), 122.9 (im), 121.8 (acr), 121.7 (acr), 54.1 (CH_2).ESI$^+$-MS, m/z: .442 [M-Cl]. Anal. Calcd (%) for $C_{23}H_{17}N_3ClAg$: C, 57.70; H, 3.58; N, 8.78. Found: C, 57.55; H, 3.32; N, 8.66

$[AgCl(CH_3\,imAcr)]$ (**3MC**). ^1H NMR (DMSO-d_6,400 MHz, 298 K) δ 8.26 (d, 2H, acr),7.90-7.82 (br, 4H, acr, im), 7.53 (br, 2H, acr), 7.33 (br, 2H, acr), 3.60 (br, 3H, CH_3). ^{13}C NMR (DMSO -d_6, 100.6 MHz, 298 K): δ 174.0 (im),148.4 (acr), 139.7 (acr), 130.8 (acr), 129.3 (acr), 127.8 (acr), 125.0 (im), 123.8 (im), 122.1 (acr), 121.8 (acr), 37.9 (CH_3). ESI$^+$-MS, m/z: .366 [M-Cl]. Anal. Calcd (%) for $C_{17}H_{13}N_3ClAg$: C, 50.71; H, 3.25; N, 10.44. Found: C, 50.88;H, 2.88; N, 10.22

$[Ag(PhCH_2imAcr)_2]BF_4$. (**2BC**). ^1H NMR (DMSO-d_6,400 MHz, 298 K): δ 8.20 (d, 2H, acr), 7.85-7.81 (m, 2H, acr, 2H, im), 7.43 (m, 2H, acr)) 7.31-7.27 (br, 1H, Ph), 7.20 (d, 2H, Ph), 7.16 (d, 2H, acr), 6.99 (d,, 2H, Ph), 4.88 (s, 2H,CH_2). ^{13}C NMR (DMSO-d_6, 100.6 MHz, 298 K) δ 181.7 (im),148.9 (acr), 139.9 (acr), 136.6 (Ph), 131.3 (acr), 129.9 (Ph), 129.2 (acr), 128.6 (Ph), 128.5 (Ph), 127.7 (acr), 126.2 (im), 123.4 (im), 122.2 (2C, acr), 54.6 (CH_2). ESI$^+$-MS, m/z: .777 [M$^+$]. Anal. Calcd (%) $C_{46}H_{34}N_6BF_4Ag$: C, 63,84% ; H,3.96 ; N, 9.71%. Found C, 63.92%; H; N 9.78.

Selected Mass Spectra:

1MC

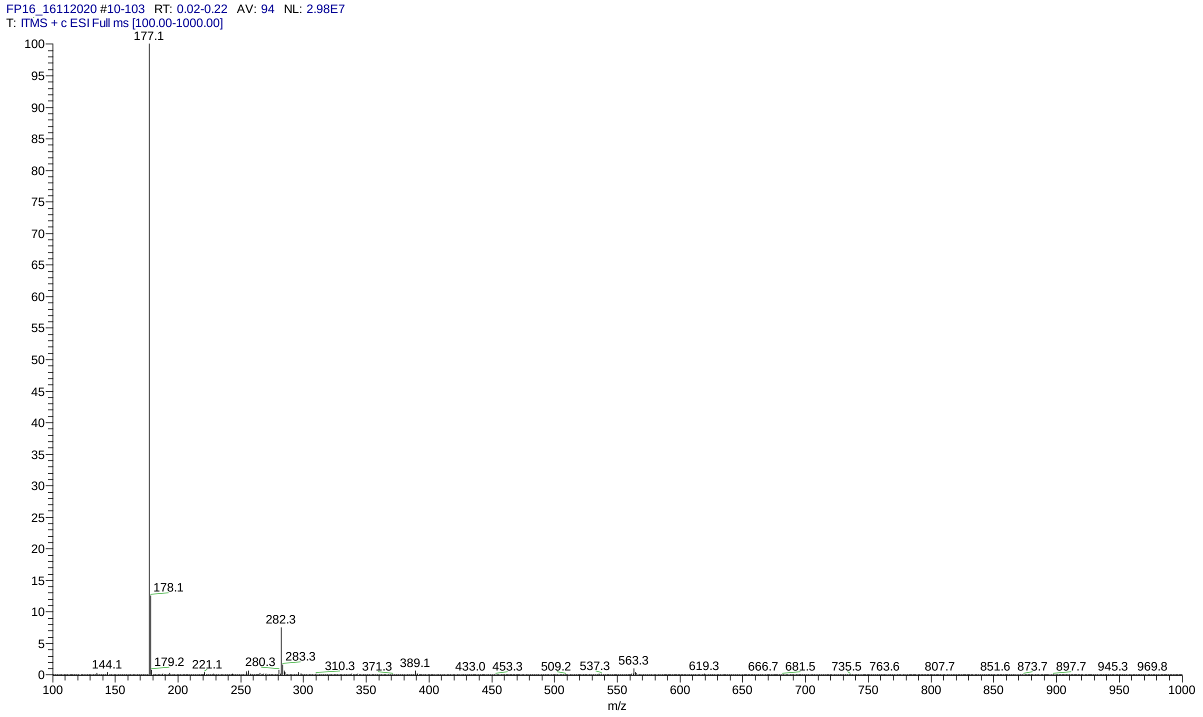

2BC

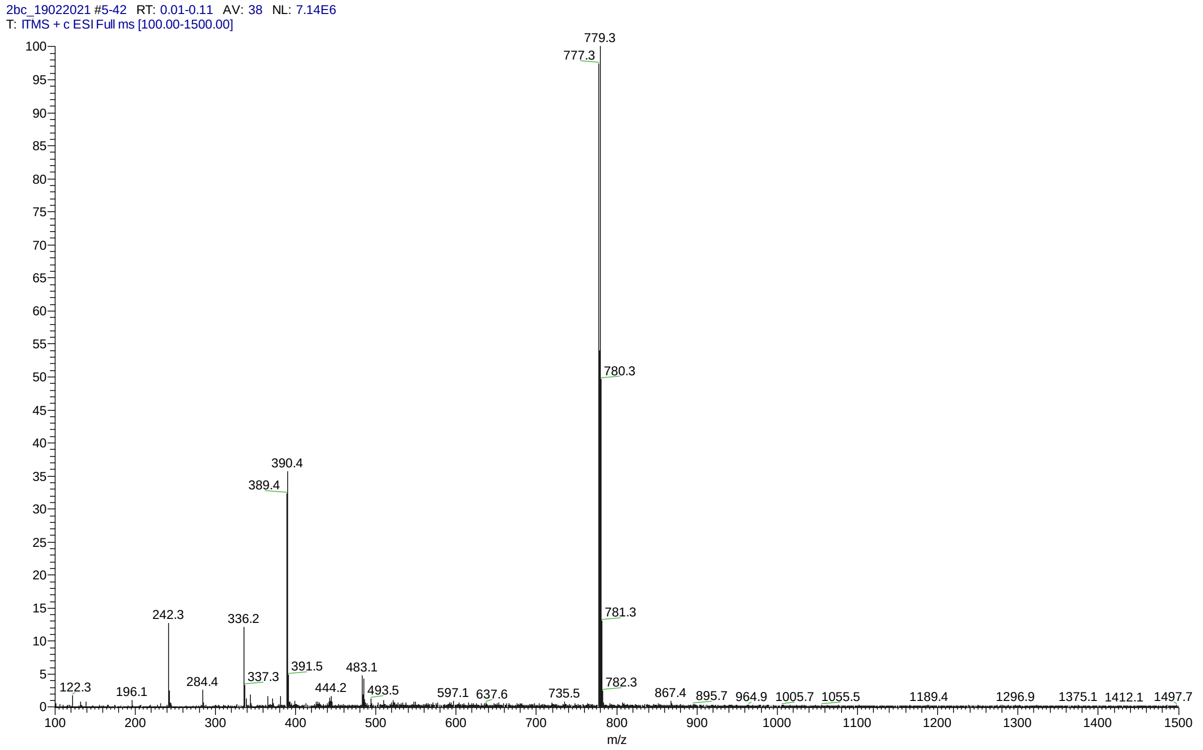

^{1}HNMR and ^{13}C NMR **1BC** Spectra

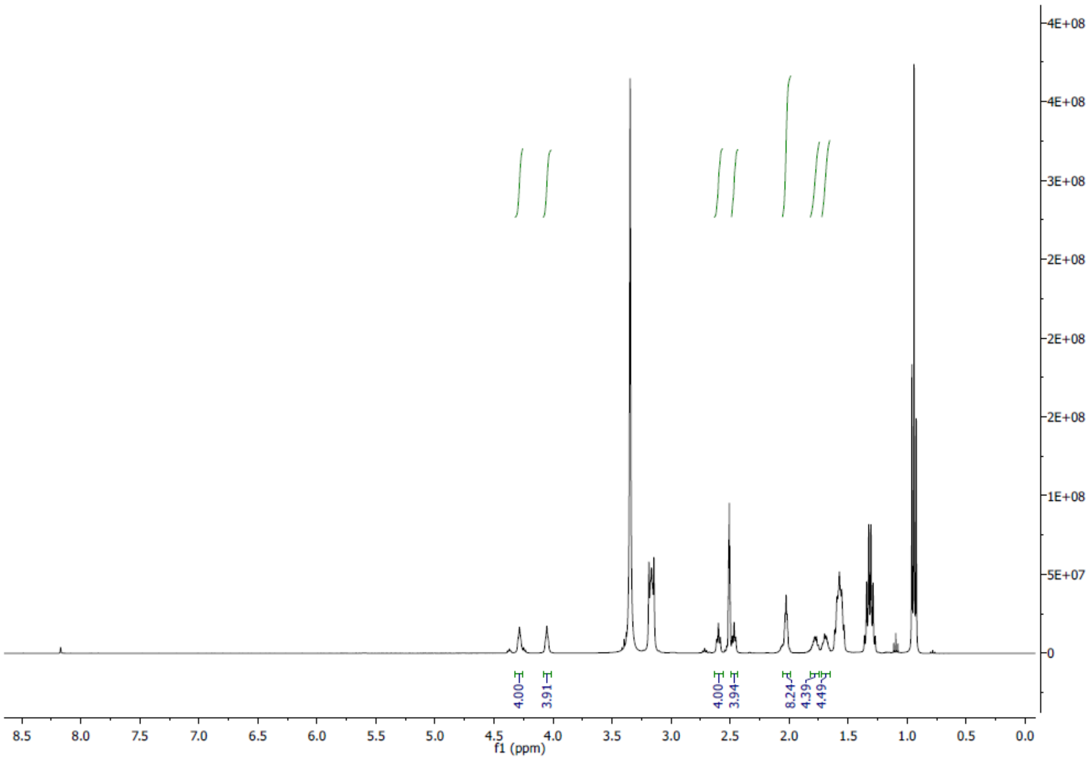

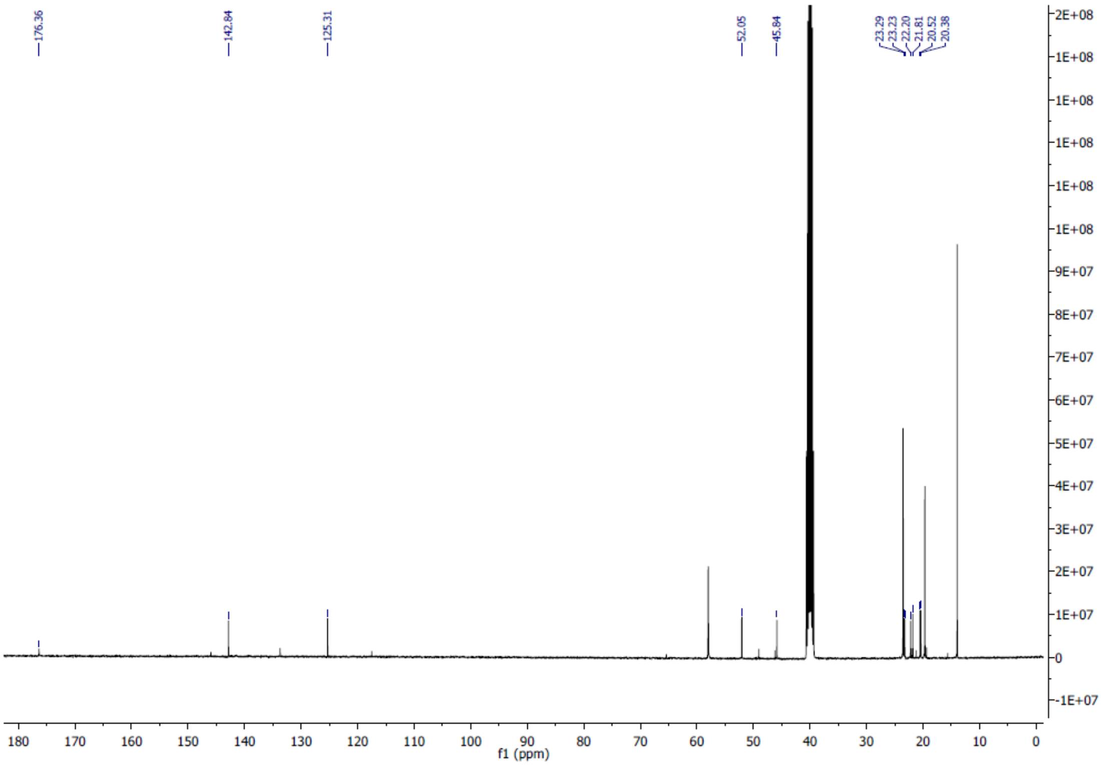

^{1}HNMR and ^{13}C NMR **1MC** Spectra

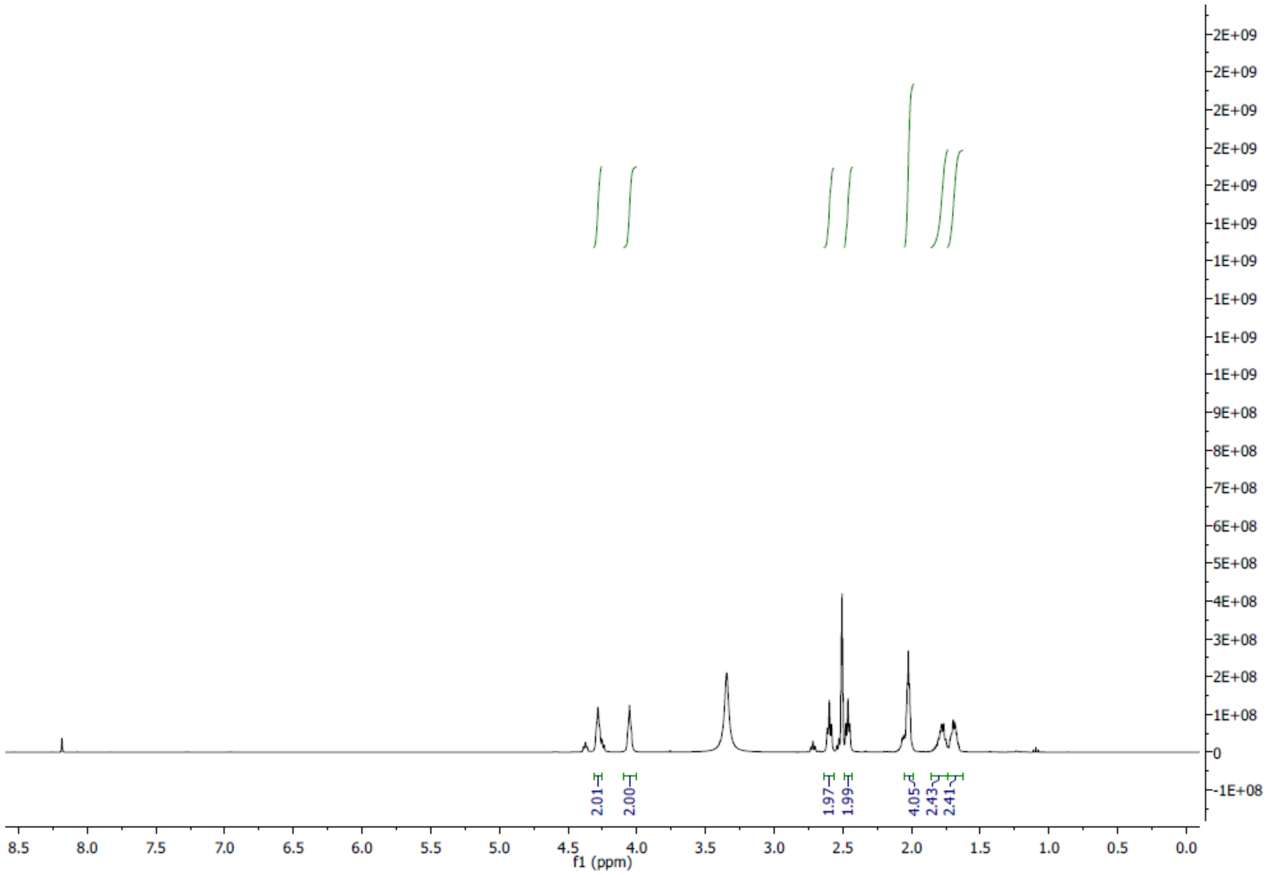

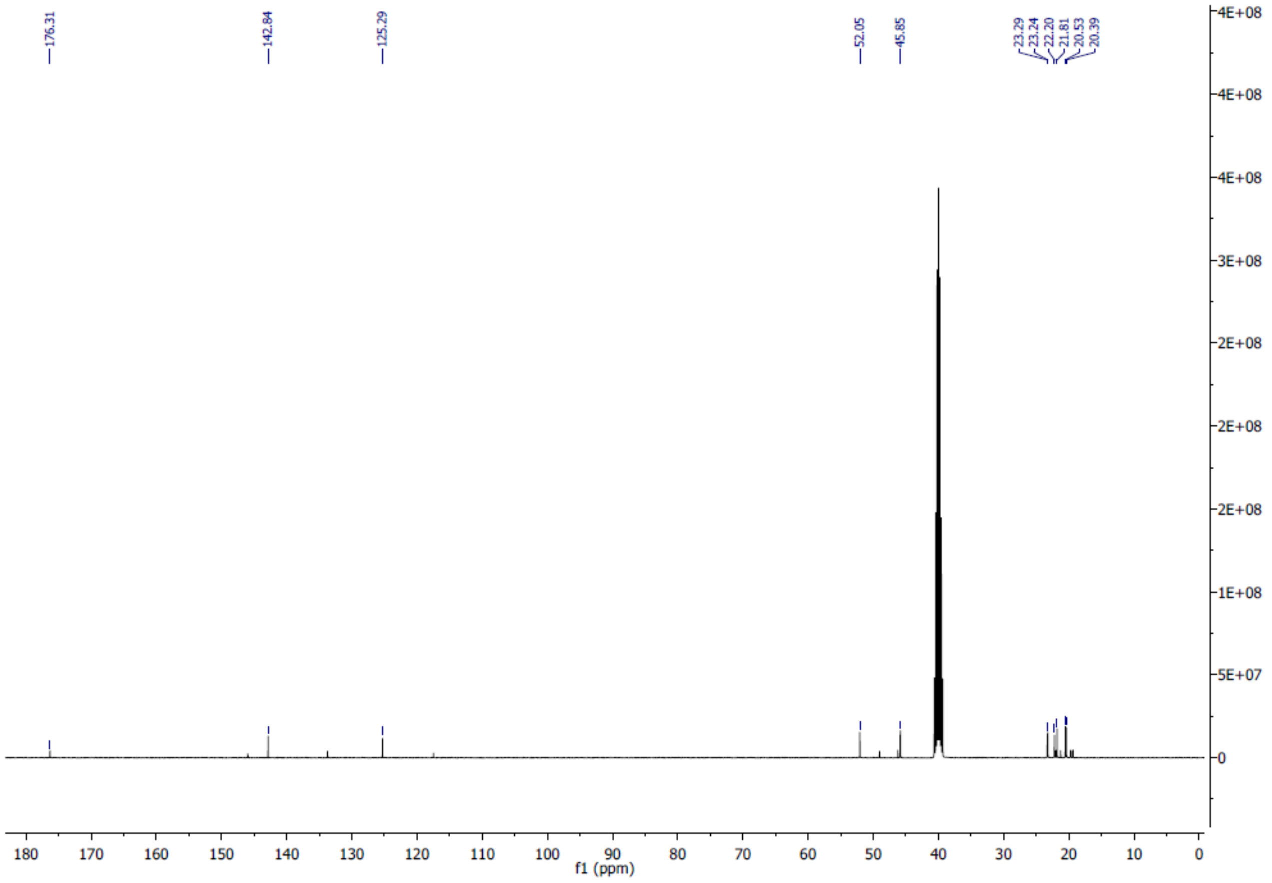

^{1}HNMR and ^{13}C NMR **2BC** Spectra

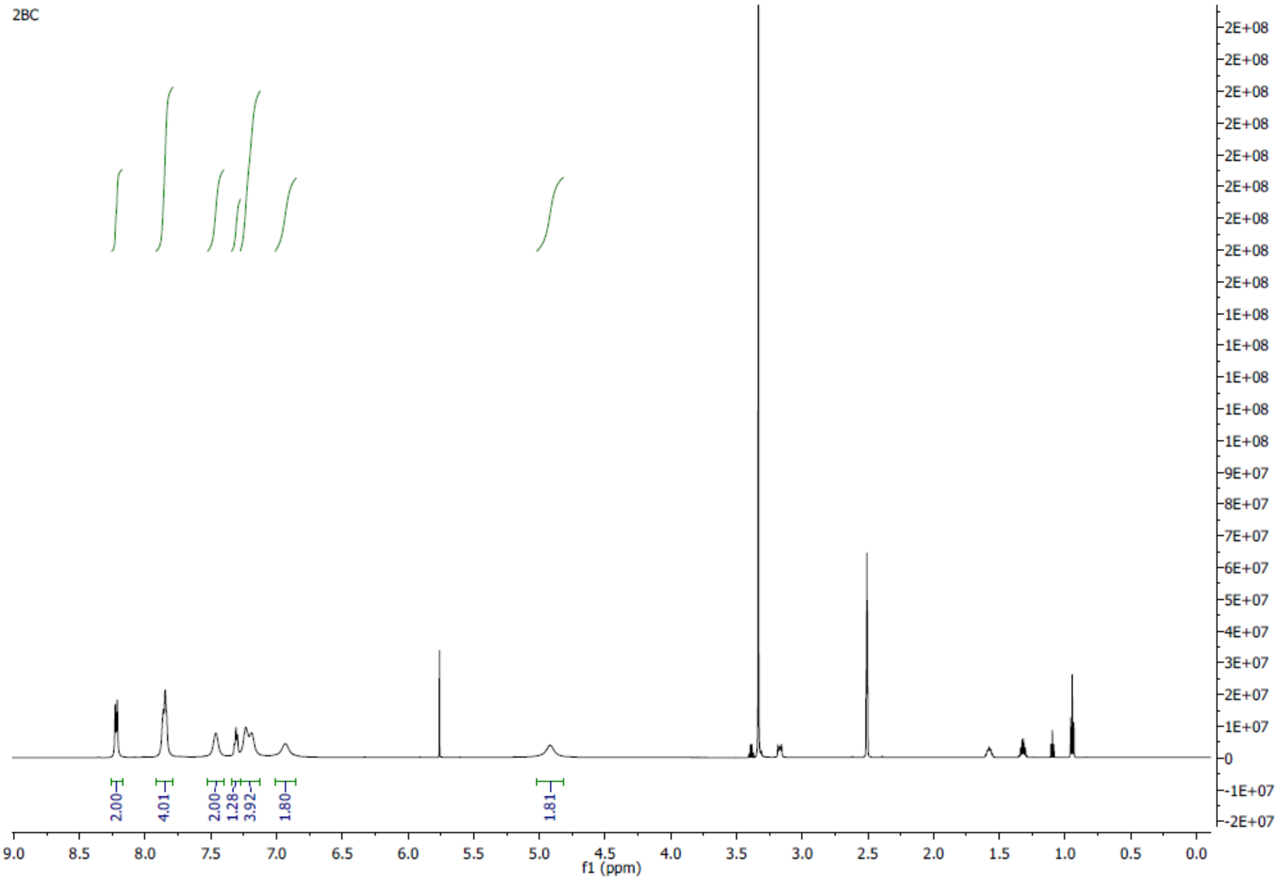

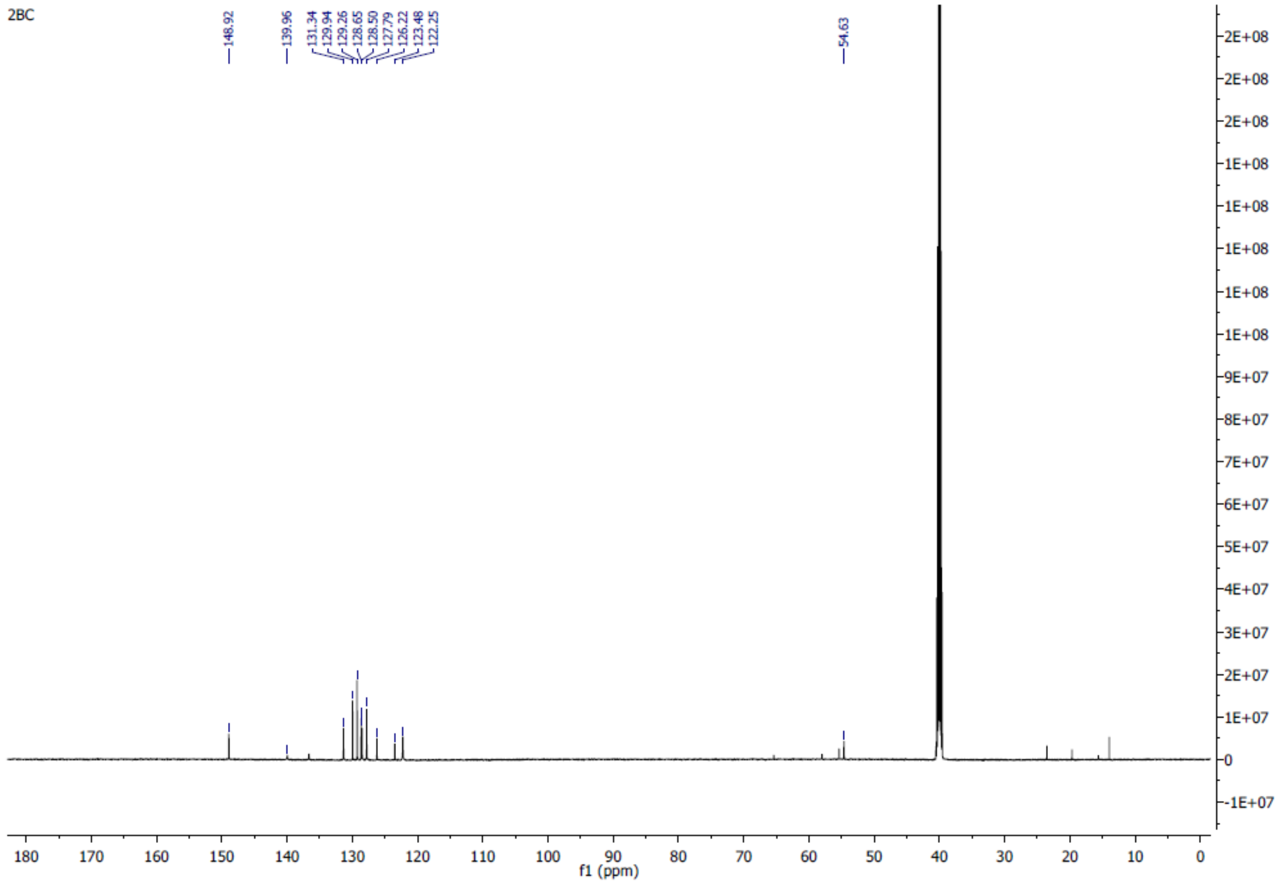

Viability test onHEK 293 cells

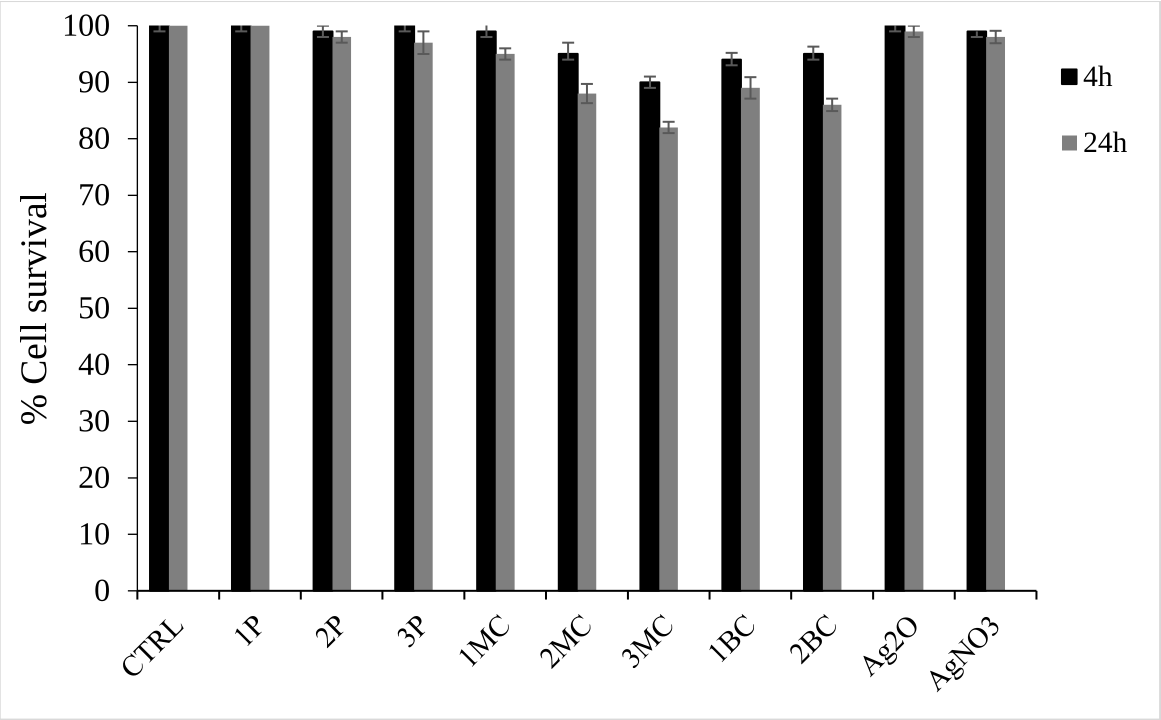

Figure S1: Cytotoxic effects of compounds [1μM] on HEK 293 cells (human embryonic kidney) by MTT assay. The mean values and SD of two independent experiments performed in triplicate are shown.
